# Modulation of host signaling and cellular responses by *Chlamydia*

**DOI:** 10.1186/1478-811X-11-90

**Published:** 2013-11-22

**Authors:** Adrian Mehlitz, Thomas Rudel

**Affiliations:** 1 University of Wuerzburg, Biocenter, Department of Microbiology, Am Hubland, D-97074, Wuerzburg, Germany

**Keywords:** *Chlamydia*, Invasion, Inclusion, Type III secretion, Tarp, Inc, Signaling, Trafficking

## Abstract

Modulation of host cell signaling and cellular functions is key to intracellular survival of pathogenic bacteria. Intracellular growth has several advantages e.g. escape from the humoral immune response and access to a stable nutrient rich environment. Growth in such a preferred niche comes at the price of an ongoing competition between the bacteria and the host as well as other microbes that compete for the very same host resources. This requires specialization and constant evolution of dedicated systems for adhesion, invasion and accommodation. Interestingly, obligate intracellular bacteria of the order *Chlamydiales* have evolved an impressive degree of control over several important host cell functions. In this review we summarize how *Chlamydia* controls its host cell with a special focus on signal transduction and cellular modulation.

## Introduction

*Chlamydia trachomatis* is an important human pathogen and the best investigated member of the order *Chlamydiales*[1]. Infection with *C. trachomatis* is among the most frequent causes of sexual transmitted diseases (STD). Infections of the upper inner eyelid eventually leading to scarring blindness (trachoma) are worldwide among the most frequently occurring ocular infections with nearly 140 million infected and 500 million at risk (source WHO). *C. pneumoniae* is a common agent of respiratory disease with sero-positivity as high as 30-45% in adults [2,3] and association with chronic diseases like arteriosclerosis or lung cancer [4,5].

*Chlamydiae* are obligate intracellular bacteria with a gram-negative atypical cell wall [6]. Growth is characterized by a distinct biphasic cycle of development. The extracellular infectious elementary bodies (EB) adhere and upon internalization into the host cell start an infectious cycle. Once internalized, EB quickly differentiate into metabolically active, dividing reticulate bodies (RB). The common perception of EB as metabolically inactive has recently been challenged by the discovery of developmental form specific metabolic requirements [7]. Adherence of an EB to the host cell is mediated by bacteria-host receptor interactions that initiate signaling via the adhesin-bound receptor and concomitantly by other bacterial effector proteins to trigger the rapid internalization of the bacteria [8-12]. Bacterial uptake leads to formation of a heavily modified pathogen containing vacuole termed inclusion [13]. Modification of the inclusion is required to prevent endolysosomal fusion and to direct acquisition of various metabolites or nutrients e.g. iron or sphingomyelin [14-17]. At the end of the infectious cycle *Chlamydia* is released from the host cell by lysis or a process that has been termed extrusion [18,19]. *Chlamydia* is able to enter a reversible persistent state through limitation of either nutrients (e.g. iron, amino acids) or application of antibiotics (e.g. penicillin) [20,21]. Persistence is characterized by formation of aberrant bodies, an incomplete developmental cycle, ongoing metabolic activity and altered gene expression [22,23]. Upon removal of the persistence inducer *Chlamydia* can reactivate and enter an acute developmental cycle.

Modulation of various host cell processes by *Chlamydia* is a prerequisite to complete the developmental cycle. Manipulation of the host cell requires specialized secretion systems e.g. the type three secretion system (TTSS) and its effector proteins and the respective genes for the TTSS can be found in all sequenced chlamydial genomes [24,25]. Other factors include e.g. the adhesins/invasins polymorphic membrane protein D (PmpD) [12,26] and outer membrane complex B (OmcB) [27]. Here, we review *Chlamydia*-induced signaling and the required bacterial effectors and sort both according to infection time and intracellular location. We apologize to all the authors, whose work could not be discussed in this review due to space constraints. For in depth reading we refer the interested reader to a recently published book [28].

### Adhesion

**Figure 1:**
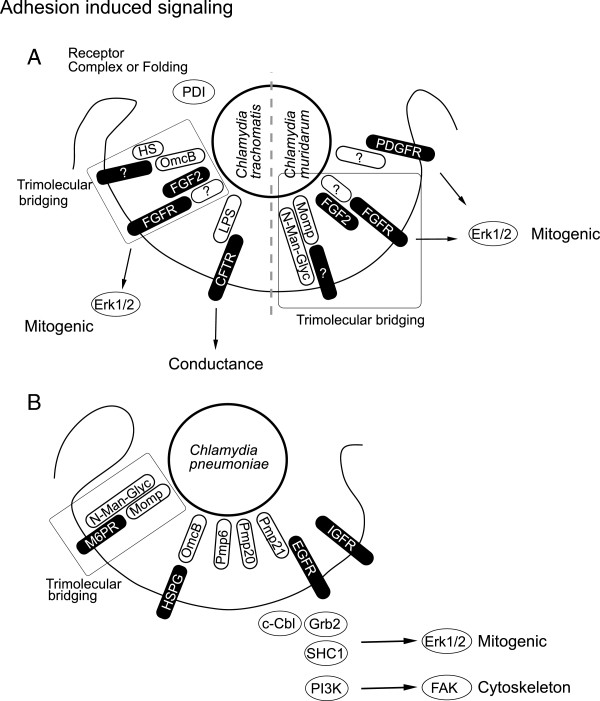
**Adhesion induced signaling. A**, Adhesin-receptor pairs are ill defined for the closely related pathogens *C. trachomatis* and *C. muridarum*. Several surface proteins like lipopolysaccharide (LPS), major outer membrane protein (MOMP), outer membrane complex B (OmcB) and polymorphic membrane protein (Pmp21) have been suggested as potential bacterial adhesins. A trimolecular bridge is thought to connect MOMP, OmcB and FGFR to their host or bacterial counterpart, respectively. Binding to host receptors like fibroblast growth factor receptor (FGFR) or platelet derived growth factor receptor (PDGFR) induces mitogenic signaling via extracellular-signal-regulated kinase 1/2 (Erk1/2). Receptor surface presentation and folding via protein disulfide isomerase (PDI) shows the necessity for specific host receptor binding. **B**, *C. pneumoniae* binds to its host cell in a bimolecular fashion via OmcB heparin sufate proteoglycan (HSPG) interaction. Binding between OmcB and HSPG is probably a reversible initial reversible binding step followed by irreversible specific binding. One adhesin receptor pair involved is Pmp21 – EGFR. The Pmp21 – EGFR interaction then triggers invasion of *Chlamydia*. Further, binding to EGFR also recruits growth factor receptor bound 2 (Grb2), Cas-Br-M (murine) ecotropic retroviral transforming sequence (c-Cbl), SHC (Src homology 2 domain containing) transforming protein 1 (SHC1) and phosphatidyl-inositol-3-kinase (PI3K) signaling, which initiates mitogenic Erk1/2 signaling as well as cytoskeletal rearrangements via focal adhesion kinase (FAK). Pmp6 and 20 have been suggested as additional adhesins on the bacterial side, while insulin growth factor receptor (IGFR) has been indicated on the host side.

Efficient adhesion to host cells is a prerequisite for invasion and intracellular life and usually requires several adhesins. *Chlamydia* has evolved a number of ways to attach to various host cells and infect different tissues according to serovariant and species [29,30]. Early research focused on the role of the abundant major outer membrane protein (MOMP) as an adhesin [31] (Figure 1A-B). Blocking the exposed variable MOMP domains using specific antibodies disturbed binding to the host cell [32]. *Chlamydia muridarum* MOMP has been described to mediate attachment to host cells as a cytoadhesin [33]. Further, MOMP from various chlamydial species is glycosylated (mainly D-mannose-rich) and this modification is critical for MOMP adhesion [34-36]. The mannose-6-phosphate / insulin-like growth factor receptor 2 (M6PR/IGFR2) has been suggested as the host receptor for MOMP, since the MOMP glycan moiety is similar to the M6PR ligand mannose-6-phosphate and blocking the M6PR prevents *C. pneumoniae* attachment and invasion [37].

Also, heparan sulfate-like glycosaminoglycan (GAG) attached to *Chlamydia* has been shown to bridge host and bacterium [38] (Figure 1A-B). Cleaving this GAG compound from the bacteria renders them non-adhesive, while addition of exogenous heparan sulfate restored attachment. GAG of a size similar to heparin or heparan sulfate has subsequently been found in the inclusion produced by *Chlamydia*[39]. Chlamydial synthesis of GAG is consistent with the observation that *C. trachomatis* also infects CHO cells deficient in heparin sulfate biosynthesis [40]. Outer membrane complex B (OmcB), a cysteine rich membrane protein, has been described to bind to GAG [41,42]. Further, GAG binding varies depending on the specific serovariant [27,43] and this binding has recently been attributed to a strain specific motif within the N-terminus of OmcB [44]. Variation in GAG binding has been suggested to co-determine cell type specificity [45].

Most of the studies performed so far on *Chlamydia*-host binding focused on bacterial adhesins and only limited data are available on the nature of host cell receptor(s). Correct surface presentation of specific host proteins has been suggested to be important using CHO cells expressing a defective protein disulfide isomerase (PDI) [46,47]. In this model PDI is most likely involved in the folding, surface presentation or receptor complex formation (Figure 1A). Attachment of *C. trachomatis* to host cells has been shown to require sulfation but no specific receptors were identified [48]. More recently, epidermal growth factor receptor (EGFR/ERBB) has been shown to be the host receptor for *C. pneumoniae* Pmp21, but not Pmp21 of *C. trachomatis*[12] (Figure 1A-B). Residual adhesion and invasion upon EGFR depletion indicates that other receptors are involved in adherence [12]. In case of *C. trachomatis,* lipopolysaccharide (LPS) has been demonstrated to be a ligand for the human cystic fibrosis transmembrane conductance regulator (CFTR) [49]. The closely related mouse pathogen *C. muridarum* engages the Fibroblast growth factor receptor (FGFR) for invasion. In this case, fibroblast growth factor 2 (FGF2) binds to *C. muridarum* and mediates invasion via FGFR [50]. The bacterial ligand for FGFR is still unknown (Figure 1A).

### Adhesion accompanied signaling

Until today a systematic approach to identify host receptors for *C. trachomatis* and *C. pneumoniae* e.g. by applying RNA interference has not been undertaken. One difficulty may be receptor redundancy that prevents the straightforward identification of receptors by single knockdowns. On the bacterial side the upcoming establishment of a genetic system just recently opened the door to systematic forward genetic searches in *Chlamydia*. We can learn a lot about bacteria-induced signaling from the recently discovered adhesin – receptor pair Pmp21 - EGFR [12]. Pmp21 coated latex beads are endocytosed in an EGFR-dependent manner demonstrating that Pmp21 is sufficient to trigger invasion [12]. The *C. trachomatis* homolog PmpD has also been implicated in adhesion, however direct experimental evidence for its function as adhesin is still missing [26]. Binding of Pmp21 to EGFR activates the receptor leading to formation of a complex with the adaptor protein growth factor receptor bound-2 (Grb2) and the ubiquitin ligase Cas-Br-M (murine) ecotropic retroviral transforming sequence (c-Cbl). EGFR activation subsequently leads to extracellular-signal-regulated kinase 1/2 (Erk1/2) activation [12] (Figure 1B). *C. pneumoniae* invasion has been shown to be accompanied by activation of src homology containing (SHC1), Erk and phosphoinositol 3 kinase (PI3K) [51]. Apparently, SHC1, Erk and PI3K activation is initiated by EGFR activation and may together lead to FAK activation (Figure 1B). Involvement of additional adhesin – receptor pair is likely and OmcB presents a strong candidate on the bacterial side because of its heparin sulphate binding domain [27,42].

**Figure 2:**
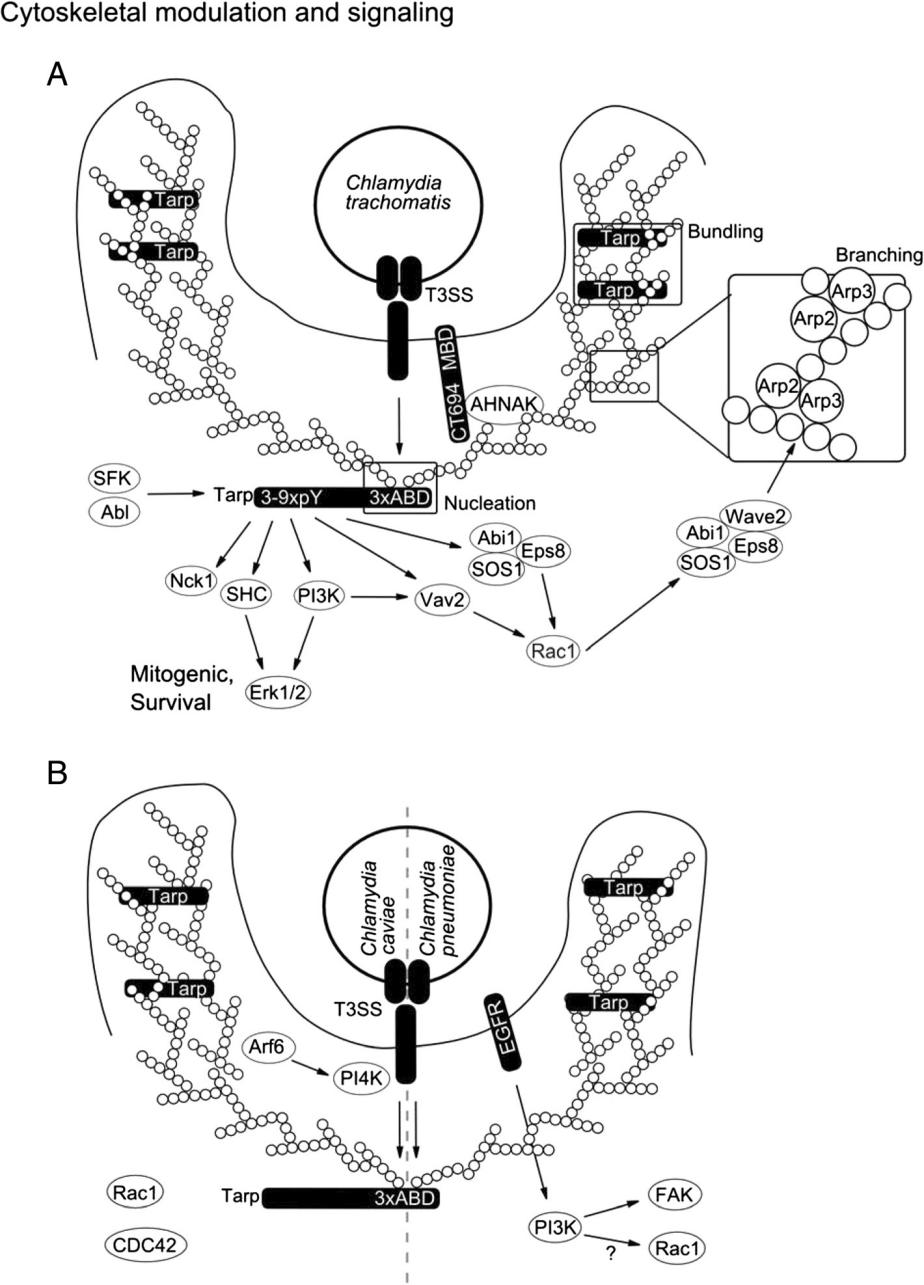
**Cytoskeletal modulation and signaling. A**, Invading *C. trachomatis* is believed to secrete a cocktail of preformed effectors into the host cell and this is supported by the two discovered factors translocated actin recruiting phosphor protein (Tarp) and CT694. Tarp initiates multiple signaling cascades, i.e. its N-terminus is phosphorylated on several tyrosine residues (pY) and the C-terminally located actin binding domains (ABD) mediate actin nucleation and bundling. Signaling via the N-terminus leads to survival signaling via extracellular-signal-regulated kinase 1/2 (Erk1/2) as well as actin branching via son of sevenless homolog 1 (SOS1) / abl-interactor 1 (Abi1) / epidermal growth factor receptor pathway substrate 8 (Eps8) / WAS protein family, member 2 (WASF2 alternative name: Wiskott-Aldrich syndrome protein family member 2 - Wave2) and actin related protein 2/3 (Arp2/3) complex. CT694 consists of a membrane binding domain (MBD) and an AHNAK nucleoprotein (AHNAK) binding region which probably links membrane to actin signaling. **B**, *C. caviae* and *pneumoniae* secrete Tarp which does not contain the N-terminal tyrosine phosphorylation domain. The C-terminal ABD motives are sufficient for actin bundling and nucleation and some of the functions linked to *C. trachomatis* Tarp might be executed via epidermal growth factor receptor (EGFR). Activation of the small GTPases ras-related C3 botulinum toxin substrate 1 (rho family, small GTP binding protein Rac1) (Rac1) and cell division cycle 42 (GTP binding protein, 25 kDa) (CDC42) has been shown for *C. caviae*, the bacterial factors mediating this are not yet found. *C. caviae* also activates ADP-ribosylation factor 6 (Arf6), which in turn activates phosphatidyl-inositol-4-kinase (PI4K) and this might take over the membrane-actin modulating function of CT694.

CFTR has been suggested as a potential host receptor for *C. trachomatis.* Binding of LPS to CFTR reduces the conductance of CFTR, however, the consequences host signaling is unclear [49] (Figure 1A). *C. muridarum* bound to host cells specifically recruits FGFR as well as platelet derived growth factor receptor (PDGFR), but not EGFR [50,52]. Activated FGFR and PDGFR lead to mitogenic signaling via Erk1/2, which might be similar to *C. pneumoniae* induced EGFR signaling. Requirements for FGF2 have also been confirmed in the human pathogenic strain *C. trachomatis* E indicating that activation of FGFR signaling might partially replace EGFR signaling during *C. trachomatis* infection [50]. Host receptors for the MOMP glycan and OmcB GAG interaction have not yet been defined. Interestingly, *C. trachomatis* receptor signaling and recruitment might be synergistic with signaling induced by the secreted bacterial protein Tarp [53]. Tarp interacts with several of the proteins recruited to the EGFR in a serovar- and phosphorylation-dependent manner [53,54]. Phosphorylation of Tarp in turn is mediated by multiple kinases most likely Src family kinases as well as Abl kinases [52,55,56] (Figure 2A).

### Cytoskeletal rearrangements

Initial studies on *Chlamydia* invasion indicated the involvement of both actin-dependent and -independent mechanisms. Invasion was suggested to take place either through phagocytosis- (actin dependent) or pinocytosis-like (actin independent) processes [57]. These observations were supported by the differential sensitivity of *C. trachomatis* serovariants towards the f-actin disrupting agent cytochalasin D [58]. However, more and more investigations focused on actin driven processes. One reason is that recruitment of actin to the invasion site was directly shown [59] and found to be dependent on a bacterial structural component, which was subsequently identified to be the translocated actin recruiting phosphoprotein (Tarp) [8,59] (Figure 2A). Tarp is synthesized during the late stages of infection and is most likely secreted into the host cell via the TTSS [8,60,61]. Surprisingly, Tarp tyrosine phosphorylation and actin recruitment are not coupled [62]. It turned out that Tarp is a nucleator of actin since it contains several actin-binding domains (ABD) with similarity to WH2 domain proteins. In addition, a proline rich region in Tarp may enhance actin oligomerization [63]. Tarp-mediated actin binding is conserved across species and is likely to be required for chlamydial invasion as invasion was blocked by anti-ABD sera [64]. Actin nucleation and bundling activities are separated in different ABD and the rate of actin polymerization is synergistic with the host Arp2/3 complex emphasizing the complexity of bacterially induced cytoskeletal modulation [65,66] (Figure 2A). Many pathogens require several cytoskeletal modulators for efficient invasion of their host cells. The chlamydial effector CT694 was discovered more recently and similarly to Tarp shows late expression and early secretion [9]. A search for cellular interaction partners identified the C-terminus of CT694 as a domain that interacts with host AHNAK and actin [9]. AHNAK is a localized to the apical plasma membrane where it interacts with actin to maintain the architecture of polarized cells [9]. In addition, AHNAK plays a role as a scaffold protein, thereby connecting protein kinase C alpha (PKCα) and phospholipase C gamma (PLCγ) signaling [9]. The N-terminus contains a membrane localization domain suggesting that CT694 functions in actin modulation during invasion [67] (Figure 2A).

Small GTPases are important modulators of actin dynamics and downstream signaling and many bacteria evolved ways to modulate host GTPases. *C. trachomatis* requires the small GTPase ras-related C3 botulinum toxin substrate 1 (Rac1) but not cell division cycle 42 (Cdc42) or ras homolog gene family member A (RhoA) for invasion [68]. Rac1 has been shown to interact with abl interactor 1 (Abi1) and WAS protein family, member 2 (WASF2; also known as Wiskott-Aldrich syndrome protein family member 2 - Wave2) in order to regulate the actin-related protein complex 2/3 (Arp2/3) and thus modulates actin recruitment and branching [69]. Activation of Rac1 might be Tarp dependent as phosphorylated Tarp interacts with the Abi1 / son of sevenless homolog 1 (SOS1) / epidermal growth factor receptor pathway substrate 8 (Eps8), vav 2 guanine nucleotide exchange factor (Vav2) and phosphoinositol 3 kinase (PI3K) upstream of Rac1 [53,54]. The requirement of GTPase for invasion differs among *Chlamydia* species as *C. caviae* needs the small GTPases Rac1 and Cdc42 but not RhoA during invasion [70] (Figure 2A-B).

Tarp from *C. caviae* does not possess the phosphorylation sites required for Rac activation; this suggests that another bacterial factor for the activation of Rac1 and/or Cdc42 exists. One pathway to Rac1 activation during *C. pneumoniae* invasion could stem from EGFR-mediated PI3K activation and it is tempting to speculate that EGFR contributes to Rac1 activation during *C. pneumoniae* infection in an analogous fashion as phosphorylated TARP does during *C. trachomatis* infection (Figure 2A-B). So far, data on the role of EGFR for *C. caviae* and Rho GTPases for *C. pneumoniae* infection are still missing, respectively. Another GTPase involved in remodeling of the actin cytoskeleton during *C. caviae* invasion is ADP ribosylation factor 6 (Arf6) [71]. Arf6 activates phosphatidylinositol 4-phosphate 5-kinase (PI4K) which is important for plasma membrane modulation during actin rearrangement, suggesting a similar function as has been proposed for CT694. A bacterial component activating Arf6 has not been described and awaits further investigation (Figure 2B).

### Establishment of the inclusion

The exact origin of the endosomal membrane is a matter of ongoing research. Caveolin [72,73], membrane rafts [73,74] and clathrin-mediated [75,76] endosome formation have been suggested as entry route for *Chlamydia*. However, these findings are still a matter of discussion since these pathways of endosome formation have not been confirmed by others [77,78]. This may be partly due to the use of different chlamydial species in these reports (*C. trachomatis* vs. *C. pneumoniae* vs. *C. caviae)* since these species differ not only in their host receptor but also in their invasion-mediated signaling. Due to these differences, varying experimental conditions had to be used e.g. for cell culture infection (centrifuge assisted vs. static). In analogy to influenza virus entry [79] and considering actin-dependent and -independent invasion mechanisms as well as differences in adhesion and entry signaling between species, a multi-route entry is likely.

**Figure 3:**
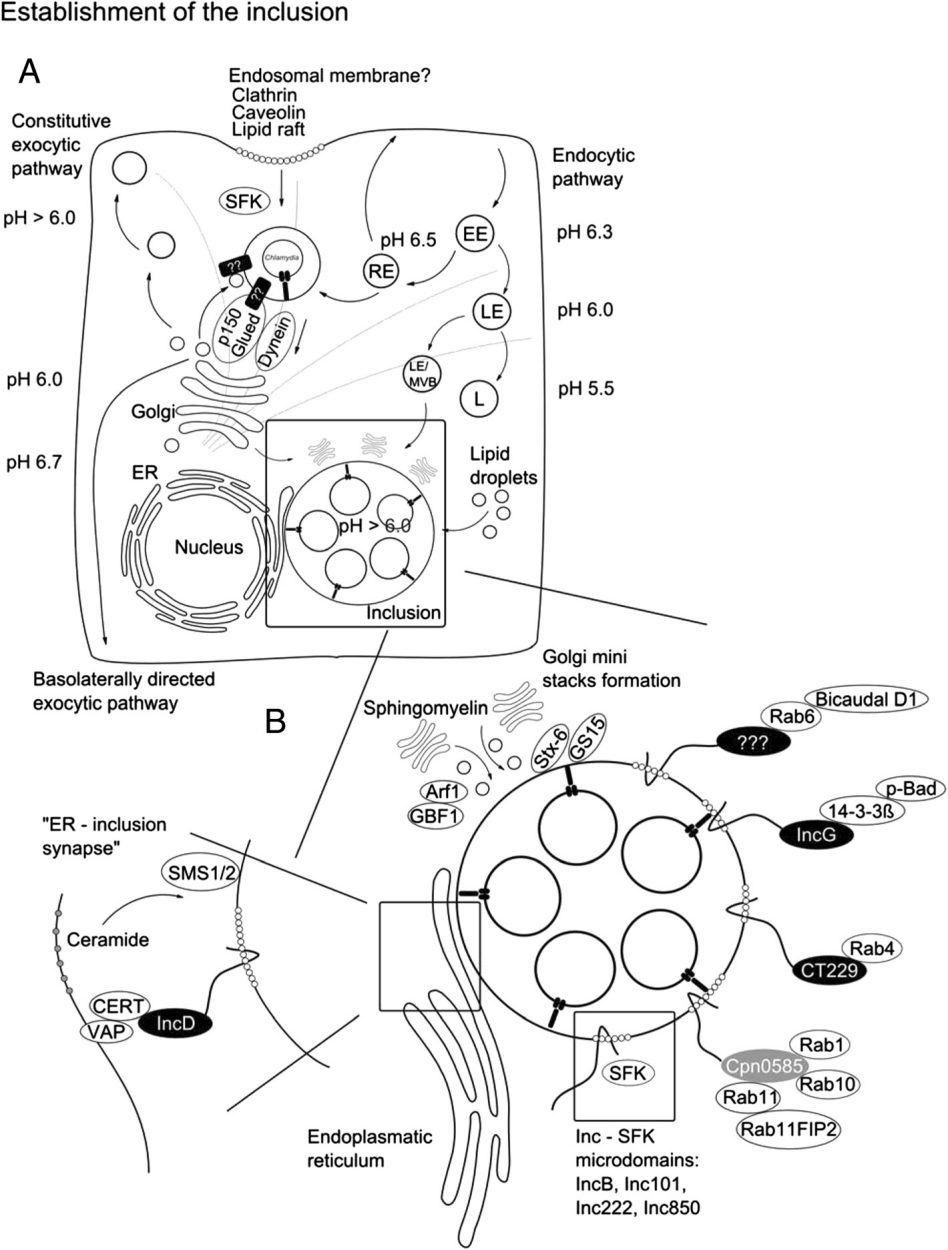
**Establishment of the inclusion. A**, The origin of the endosomal membrane of *Chlamydia* is not yet solved. Entry via multiple routes was suggested e.g. clathrin, caveolin or lipid raft dependent. After invasion early inclusions deviate from the endosomal route and acquires sphingolipids from the basolaterally directed exocytic route. Bacterial factors directing the exocytic trafficking towards the inclusion are not yet known. Similar to the Golgi (the most important inclusion lipid source) the inclusion pH stays above 6.0. Other lipid sources are via CD63+ late endosomes/ multivesicular body (LE/MVB), lipid droplets and through recruitment of the high density lipoprotein (HDL) synthesis machinery. EE (Early endosome), LE (Late endosome), RE (Recycling endosome) and L (Lysosomes). Transport of the inclusion to the microtubule organizing center (MTOC) requires Src family kinases (SFK), dynein, the dynactin subunit p150 Glued and an unidentified bacterial factor. Positioning and growth of the inclusion is accompanied by Golgi ministack formation in *C. trachomatis*. **B**, Interaction with various cellular organelles is mediated via a wide set of inclusion membrane proteins. IncG, CT229 and Cpn0585 are among the best investigated Inc proteins shown to interact with host proteins. Apoptosis is controlled via IncG/14-3-3 beta/ BCL2-associated agonist of cell death (p-Bad) interaction. Organelle identity is probably mediated via CT229 which interacts with Rab4 and Cpn0585 (interacts with Rab1, 10 and 11). Moreover, Incs segregate into micro domains and this is often associated with SFK co-localization. ER – inclusion synapses have been suggested to be an additional routes of lipid uptake. This is mediated via IncD/ collagen, type IV, alpha 3 (Goodpasture antigen) binding protein (CERT) / VAMP (vesicle-associated membrane protein)-associated protein A, 33 kDa (VAP-A) interaction. CERT is a lipid carrier suggested to transfer ceramide into the inclusion membrane where ceramide is converted to sphingomyelin via sphingomyelin synthase 1/2 (SMS1/2).

Beside the ongoing discussion on the endosomal origin in *Chlamydia* infection consensus exists that once the endosome is formed it quickly separates from the endosomal route and starts to acquire sphingomyelin from the exocytic route [17,80] (Figure 3). Vacuolar pH stays above 6.0 indicating that lysosomal fusion is prevented [81]. Acquisition of sphingomyelin and prevention of lysosomal degradation require bacterial components since inhibition of bacterial transcription and translation interfere with these processes [82]. Interestingly, *Chlamydia* may use preformed early secreted or surface presented effectors to prevent lysosomal degradation as lysosomal maturation is delayed even in the presence of bacterial translation inhibitors [83]. Only a limited number of early chlamydial effectors have yet been characterized. Tarp and CT694, two of these effectors involved in actin modulation have been discussed in the previous section. A recent report describes ChlaOTU as another early effector with deubiquitinating activity [84]. Formation of endosomes with *C. caviae* is accompanied by extensive ubiquitination, which is likely removed through the action of ChlaOTU. Interaction between ChlaOTU and host autophagy receptor NDP52 has been observed but appears to be dispensable for infection [84]. ChlaOTU is well conserved in *C. pneumoniae* but homology in *C. trachomatis* and *C. muridarum* is weak [84]. Transport of early inclusions of *C. trachomatis* and *C. pneumoniae* proceeds in a microtubule and Src family kinase dependent manner resulting in transport to the microtubule organizing center (MTOC) [85-87]. Interestingly, inclusions of the nonhuman chlamydial species *C. caviae* and *C. muridarum* are not transported to the MTOC [87]. Transport to the MTOC requires host cell vesicle transport and is dynein dependent but p50 dynamitin independent, as was shown by microinjection of antibodies against these proteins [86]. Antibodies directed against the plus end motor protein kinesin did not affect the transport while p150 (Glued) (subunit of the dynactin complex) co-localized to the endosome. The absence of p50 dynamitin which links vesicular cargo to dynein suggests that a bacterial factor within the endosomal membrane exerts this function [86]. During transport to the MTOC, the *Chlamydia*-containing endosome quickly deviates from the endosomal route, i.e. it is negative for endosomal fluid phase as well as lysosomal markers [88,89]. The exocytic Golgi to plasma membrane pathway is interrupted and *Chlamydia*-harboring endosome aquires sphingomyelin [17,80]. Interruption of Golgi derived exocytic transport might require the manipulation of small Rab GTPases, e.g. it has been shown that sphingomyelin acquisition is controlled by Rab14 around 10 hours post infection [90]. It remains to be investigated whether this process is controlled via interaction with early-secreted bacterial proteins, however, most of the investigated small Rab GTPases are recruited to the maturing inclusion [91]. Rab GTPases are selectively recruited in a species-dependent and -independent manner, probably through interaction with inclusion membrane proteins [91]. Selective recruitment of Rab GTPases regulates the interaction with various host organelles and this is supported by recruitment of several Rab interactors e.g. Bicaudal D1 (Rab 6 interactor), oculocerebrorenal syndrome of Lowe (OCRL1, interacts with multiple Rabs) and RAB11 family interacting protein 2 (Rab11FIP2, Rab11 and 14 interactor) [92-94]. Intracellular development of the inclusion is accompanied by extensive lipid acquisition from various sources. One of the major lipid sources appears to be the Golgi apparatus [17,95,96] that is fragmented during *C. trachomatis* infection probably to facilitate lipid transport to the inclusion [97]. Fragmentation of the Golgi and ceramide acquisition has been suggested to depend on Rab6/11 [98] and this process might be specific for *C. trachomatis* as it was not yet described for any other chlamydial species. The Golgi as the major lipid source is supported by preferential interception of basolaterally directed Golgi derived exocytic vesicles and recruitment of the trans-Golgi Snare syntaxin 6 (STX6) to the inclusion [99,100]. In line with this, *Chlamydia* intercepts retrograde intra-Golgi trafficking through recruitment of GS15 positive Conserved Oligomeric Golgi (COG) complex vesicles [101]. Additionally, optimal growth requires control of lipid trafficking from CD63-positive late endocytic multivesicular bodies, acquisition of cytoplasmic lipid droplets as well as recruitment of the high density lipoprotein (HDL) biogenesis machinery [102-104]. Recent results obtained for *C. muridarum* indicate that sphingomyelin acquisition might proceed in both vesicle-dependent as well as independent manner [105]. Vesicular trafficking via ADP-ribosylation factor 1 (Arf1) and Golgi-specific brefeldin A resistance factor 1 (GBF1) was found to be mainly required for inclusion membrane growth and stability but not for bacterial replication. Conversely, vesicular independent transport via the lipid carrier ceramide transfer protein (CERT) which is involved in endoplasmic reticulum (ER) to trans-Golgi transport as well as acquisition of VAMP (vesicle-associated membrane protein)-associated protein A (VAP-A), sphingomyelin synthase 1 and 2 (SMS1 and 2) to the inclusion are required for bacterial replication [105]. The situation appears to be more complex as various trafficking pathways regulate sphingolipid acquisition [99,106]. Elucidating the complexity of trafficking and lipid acquisition may require the establishment of fully polarized infection models for *Chlamydia* infection.

### Contact area - proteins in the inclusion membrane

Upon completion of invasion chlamydial proteins heavily modify the endosomal membrane. Bacterial proteins present in the membrane of the inclusion and directed towards the cytoplasmic face of the inclusion membrane are likely to mediate early (positioning), mid (organelle fusion, survival control) and late (survival control, egress) effects and thereby critically regulate replication. More than 50 chlamydial proteins were detected in the inclusion membrane by immunofluorescence microscopy using specific antibodies [107]. A characteristic feature of the inclusion membrane (Inc) proteins [15] is a large hydrophobic bi-lobed transmembrane region which is useful for the *in silico* prediction of Inc proteins throughout the order of *Chlamydiales*[108-113]. Inc proteins share little sequence identity with each other, are unique for the order *Chlamydiales* and represent between 7-10% of the respective species proteomes [113]. Secretion of Inc proteins has been suggested to be TTSS dependent and this has been confirmed in a heterologous *Shigella* and *Yersinia* systems as well as by using chemical inhibitors of TTSS [114,115].

IncA is so far the best characterized Inc protein and has been shown to mediate inclusion fusogenicity through interaction of its soluble N-ethylmaleimide-sensitive-factor attachment receptor (SNARE) like cytoplasmic coiled-coil domains forming tetramer bundles [116-119]. Propagation of *C. trachomatis* was dramatically reduced in the presence of TTSS inhibitors and treatment of infected cells with TTSS inhibitors prevented translocation of IncA as well as inclusion fusion [120]. IncA homotypic interaction might be the exception rather than the rule among Inc proteins and more recent data suggest additional interactions with host SNARE proteins [119]. Host proteins have been identified as interaction partners for many of the investigated Inc proteins that could explain how *Chlamydia* modulates host cell physiology. Interaction of IncG and host 14-3-3 beta was the first described example, which was later linked to the recruitment of phosphorylated host Bcl-2-associated agonist of cell death (Bad) and indicated to be one part of chlamydial interference with apoptosis signaling [119,121] (Figure 3). Recently, Inc proteins were identified as regulators of species-specific Rab GTPase inclusion recruitment [91]. CT229 was found to interact with Rab4, while Cpn0585 interacts with Rab1, 10 and 11 [122,123]. Thus, recruitment of Rab GTPases via Inc proteins could explain inclusion-mediated regulation and control of vesicular trafficking inside the eukaryotic host cell. A subset of Inc proteins, i.e. IncB, Inc101, Inc222 and Inc850 have been shown to associate with active Src family kinases (SFK) in micro-domains and this regulates interaction with the microtubule network and maybe even SFK-mediated sphingolipid acquisition [106,124]. IncD interaction with CERT represents another example of how *Chlamydia* exerts control over sphingolipid acquisition and suggests a bridging function at inclusion ER junction sites between IncD, CERT and VAPs [125,126].

Finally, exit mechanisms might also be governed through Inc interactions as shown for the interaction between CT228 and Myosin phosphatase-targeting subunit 1 (MYPT1) [127]. Chlamydial host cell exit takes place either through a series of cysteine protease mediated proteolytic steps or extrusion, which describes an actin, N-Wasp, Myosin-II and Rho GTPase-dependent exit mechanism [18]. Both, the active as well as inactive forms of MYPT1 were recruited to the inclusion membrane. Phosphorylated inactive MYPT1 co-localized in SFK micro domains with myosin light chain 2 (MLC2), myosin light chain kinase (MLCK), myosin IIA and B. Inactivation of either MLC2, MLCK, myosin IIA or B reduced chlamydial extrusion; thus, the suggested role of CT228 mediated MYPT1 regulation is a shift of exit mechanism in response to certain environmental stimuli [127]. These examples suggest that understanding the function of chlamydial Inc and host protein complexes will be key for a deeper understanding on the mechanism how *Chlamydia* modulates the host cell. This assumption asks for a systematic investigation of Inc proteins and inclusion membrane content.

### Future directions

Due to the unique intracellular lifestyle in a membrane-bound vacuolar environment, *Chlamydia spp.* have to exploit various routes of invasion and mechanisms to maintain their niche. Here, we have summarized how *Chlamydia* modulates cellular signaling and membrane trafficking. It is apparent that significant effort is required to fully understand how *Chlamydia* occupies its niche. Some of the open tasks are e.g. identification of the adhesin host receptor repertoire, clarification of the first steps of invasion, species specificity, infection of polarized epithelial cells and transfer into *in vivo* models. Further, although the number of proteins interacting with the bacterial factors is constantly growing, functional analysis of these interactions is still in its infancy and awaiting the full use of the newly developed chlamydial genetics. Applying the power of forward genetic approaches will help to identify bacterial effectors that orchestrate the complex chlamydial adaptation in its unique niche inside the host cell.

## Abbreviations

EB: Elementary bodies; RB: Reticulate bodies.

## Competing interests

The authors declare that they have no competing interests.

## Authors’ contributions

AM and TR: drafted and revised the manuscript. Both authors read and approved the final manuscript.

## References

[B1] Everett KD Bush RM Andersen AA Emended description of the order *Chlamydiales*, proposal of *Parachlamydiaceae* fam. nov. and *Simkaniaceae* fam. nov., each containing one monotypic genus, revised taxonomy of the family *Chlamydiaceae*, including a new genus and five new species, and standards for the identification of organisms Int J Syst Bacteriol 1999 49 Pt 2 415 440 10319462 10.1099/00207713-49-2-415

[B2] Thom DH Grayston JT Wang SP Kuo CC Altman J Chlamydia pneumoniae strain TWAR, Mycoplasma pneumoniae, and viral infections in acute respiratory disease in a university student health clinic population Am J Epidemiol 1990 132 248 256 2372005 10.1093/oxfordjournals.aje.a115654

[B3] Campbell LA Kuo CC Wang SP Grayston JT Serological response to Chlamydia pneumoniae infection J Clin Microbiol 1990 28 1261 1264 2380354 10.1128/jcm.28.6.1261-1264.1990 PMC267915

[B4] Littman AJ Jackson LA Vaughan TL Chlamydia pneumoniae and lung cancer: epidemiologic evidence Cancer Epidemiol Biomarkers Prev 2005 14 773 778 10.1158/1055-9965.EPI-04-0599 15824142

[B5] Mahony JB Coombes BK Chlamydia pneumoniae and atherosclerosis: does the evidence support a causal or contributory role? FEMS Microbiol Lett 2001 197 1 9 10.1111/j.1574-6968.2001.tb10574.x 11287138

[B6] Henrichfreise B Schiefer A Schneider T Nzukou E Poellinger C Hoffmann TJ Johnston KL Moelleken K Wiedemann I Pfarr K Functional conservation of the lipid II biosynthesis pathway in the cell wall-less bacteria Chlamydia and Wolbachia: why is lipid II needed? Mol Microbiol 2009 73 913 923 10.1111/j.1365-2958.2009.06815.x 19656295

[B7] Omsland A Sager J Nair V Sturdevant DE Hackstadt T Developmental stage-specific metabolic and transcriptional activity of Chlamydia trachomatis in an axenic medium Proc Natl Acad Sci U S A 2012 109 19781 19785 10.1073/pnas.1212831109 23129646 PMC3511728

[B8] Clifton DR Fields KA Grieshaber SS Dooley CA Fischer ER Mead DJ Carabeo RA Hackstadt T A chlamydial type III translocated protein is tyrosine-phosphorylated at the site of entry and associated with recruitment of actin Proc Natl Acad Sci U S A 2004 101 10166 10171 10.1073/pnas.0402829101 15199184 PMC454183

[B9] Hower S Wolf K Fields KA Evidence that CT694 is a novel Chlamydia trachomatis T3S substrate capable of functioning during invasion or early cycle development Mol Microbiol 2009 72 1423 1437 10.1111/j.1365-2958.2009.06732.x 19460098 PMC2997736

[B10] Markham AP Jaafar ZA Kemege KE Middaugh CR Hefty PS Biophysical characterization of Chlamydia trachomatis CT584 supports its potential role as a type III secretion needle tip protein Biochemistry 2009 48 10353 10361 10.1021/bi901200y 19769366 PMC4285778

[B11] Stone CB Bulir DC Emdin CA Pirie RM Porfilio EA Slootstra JW Mahony JB Chlamydia pneumoniae CdsL regulates CdsN ATPase activity, and disruption with a peptide mimetic prevents bacterial invasion Front Microbiol 2011 2 21 21687413 10.3389/fmicb.2011.00021 PMC3109343

[B12] Molleken K Becker E Hegemann JH The Chlamydia pneumoniae invasin protein Pmp21 recruits the EGF receptor for host cell entry PLoS Pathog 2013 9 e1003325 10.1371/journal.ppat.1003325 23633955 PMC3635982

[B13] Scidmore-Carlson MA Shaw EI Dooley CA Fischer ER Hackstadt T Identification and characterization of a Chlamydia trachomatis early operon encoding four novel inclusion membrane proteins Mol Microbiol 1999 33 753 765 10.1046/j.1365-2958.1999.01523.x 10447885

[B14] Al-Younes HM Rudel T Brinkmann V Szczepek AJ Meyer TF Low iron availability modulates the course of Chlamydia pneumoniae infection Cell Microbiol 2001 3 427 437 10.1046/j.1462-5822.2001.00125.x 11422085

[B15] Rockey DD Heinzen RA Hackstadt T Cloning and characterization of a Chlamydia psittaci gene coding for a protein localized in the inclusion membrane of infected cells Mol Microbiol 1995 15 617 626 7783634 10.1111/j.1365-2958.1995.tb02371.x

[B16] Ojcius DM Hellio R Dautry-Varsat A Distribution of endosomal, lysosomal, and major histocompatability complex markers in a monocytic cell line infected with Chlamydia psittaci Infect Immun 1997 65 2437 2442 9169786 10.1128/iai.65.6.2437-2442.1997 PMC175338

[B17] Hackstadt T Rockey DD Heinzen RA Scidmore MA Chlamydia trachomatis interrupts an exocytic pathway to acquire endogenously synthesized sphingomyelin in transit from the Golgi apparatus to the plasma membrane EMBO J 1996 15 964 977 8605892 PMC449991

[B18] Hybiske K Stephens RS Mechanisms of host cell exit by the intracellular bacterium Chlamydia Proc Natl Acad Sci U S A 2007 104 11430 11435 10.1073/pnas.0703218104 17592133 PMC2040915

[B19] Chin E Kirker K Zuck M James G Hybiske K Actin recruitment to the Chlamydia inclusion is spatiotemporally regulated by a mechanism that requires host and bacterial factors PLoS One 2012 7 e46949 10.1371/journal.pone.0046949 23071671 PMC3469565

[B20] Wyrick PB Chlamydia trachomatis persistence in vitro: an overview J Infect Dis 2010 201 Suppl 2 S88 S95 20470046 10.1086/652394 PMC2878585

[B21] Hogan RJ Mathews SA Mukhopadhyay S Summersgill JT Timms P Chlamydial persistence: beyond the biphasic paradigm Infect Immun 2004 72 1843 1855 10.1128/IAI.72.4.1843-1855.2004 15039303 PMC375192

[B22] Belland RJ Zhong G Crane DD Hogan D Sturdevant D Sharma J Beatty WL Caldwell HD Genomic transcriptional profiling of the developmental cycle of Chlamydia trachomatis Proc Natl Acad Sci U S A 2003 100 8478 8483 10.1073/pnas.1331135100 12815105 PMC166254

[B23] Maurer AP Mehlitz A Mollenkopf HJ Meyer TF Gene expression profiles of Chlamydophila pneumoniae during the developmental cycle and iron depletion-mediated persistence PLoS Pathog 2007 3 e83 10.1371/journal.ppat.0030083 17590080 PMC1894823

[B24] Peters J Wilson DP Myers G Timms P Bavoil PM Type III secretion a la Chlamydia Trends Microbiol 2007 15 241 251 10.1016/j.tim.2007.04.005 17482820

[B25] Beeckman DS Vanrompay DC Bacterial secretion systems with an emphasis on the chlamydial Type III secretion system Curr Issues Mol Biol 2010 12 17 41 19605938

[B26] Wehrl W Brinkmann V Jungblut PR Meyer TF Szczepek AJ From the inside out–processing of the Chlamydial autotransporter PmpD and its role in bacterial adhesion and activation of human host cells Mol Microbiol 2004 51 319 334 10.1046/j.1365-2958.2003.03838.x 14756775

[B27] Moelleken K Hegemann JH The Chlamydia outer membrane protein OmcB is required for adhesion and exhibits biovar-specific differences in glycosaminoglycan binding Mol Microbiol 2008 67 403 419 18086188 10.1111/j.1365-2958.2007.06050.x PMC2229832

[B28] Tan M Bavoil PM Intracellular Pathogens I: Chlamydiales 2012 Washington, D.C.: ASM Press

[B29] Campbell LA Kuo CC Interactions of *chlamydia* with the host cells that mediate attachment and uptake. *Chlamydia* genomics and pathogenesis Horizon Bioscience 2006 1 505 522

[B30] Moelleken K Hegemann JH Tan M, Bavoil PM Chlamydial adhesion and adhesins Intracellular Pathogens I: Chlamydiales 2012 Washington, D.C.: ASM Press

[B31] Su H Watkins NG Zhang YX Caldwell HD Chlamydia trachomatis-host cell interactions: role of the chlamydial major outer membrane protein as an adhesin Infect Immun 1990 58 1017 1025 2318528 10.1128/iai.58.4.1017-1025.1990 PMC258576

[B32] Su H Caldwell HD In vitro neutralization of Chlamydia trachomatis by monovalent Fab antibody specific to the major outer membrane protein Infect Immun 1991 59 2843 2845 1713202 10.1128/iai.59.8.2843-2845.1991 PMC258096

[B33] Su H Raymond L Rockey DD Fischer E Hackstadt T Caldwell HD A recombinant Chlamydia trachomatis major outer membrane protein binds to heparan sulfate receptors on epithelial cells Proc Natl Acad Sci U S A 1996 93 11143 11148 10.1073/pnas.93.20.11143 8855323 PMC38298

[B34] Swanson AF Kuo CC Evidence that the major outer membrane protein of Chlamydia trachomatis is glycosylated Infect Immun 1991 59 2120 2125 1645328 10.1128/iai.59.6.2120-2125.1991 PMC257975

[B35] Swanson AF Kuo CC Binding of the glycan of the major outer membrane protein of Chlamydia trachomatis to HeLa cells Infect Immun 1994 62 24 28 8262634 10.1128/iai.62.1.24-28.1994 PMC186062

[B36] Kuo C Takahashi N Swanson AF Ozeki Y Hakomori S An N-linked high-mannose type oligosaccharide, expressed at the major outer membrane protein of Chlamydia trachomatis, mediates attachment and infectivity of the microorganism to HeLa cells J Clin Invest 1996 98 2813 2818 10.1172/JCI119109 8981929 PMC507748

[B37] Puolakkainen M Kuo CC Campbell LA Chlamydia pneumoniae uses the mannose 6-phosphate/insulin-like growth factor 2 receptor for infection of endothelial cells Infect Immun 2005 73 4620 4625 10.1128/IAI.73.8.4620-4625.2005 16040974 PMC1201205

[B38] Zhang JP Stephens RS Mechanism of C. trachomatis attachment to eukaryotic host cells Cell 1992 69 861 869 10.1016/0092-8674(92)90296-O 1591780

[B39] Rasmussen-Lathrop SJ Koshiyama K Phillips N Stephens RS Chlamydia-dependent biosynthesis of a heparan sulphate-like compound in eukaryotic cells Cell Microbiol 2000 2 137 144 10.1046/j.1462-5822.2000.00039.x 11207570

[B40] Stephens RS Poteralski JM Olinger L Interaction of Chlamydia trachomatis with mammalian cells is independent of host cell surface heparan sulfate glycosaminoglycans Infect Immun 2006 74 1795 1799 10.1128/IAI.74.3.1795-1799.2006 16495553 PMC1418640

[B41] Stephens RS Koshiyama K Lewis E Kubo A Heparin-binding outer membrane protein of chlamydiae Mol Microbiol 2001 40 691 699 10.1046/j.1365-2958.2001.02418.x 11359574

[B42] Fadel S Eley A Chlamydia trachomatis OmcB protein is a surface-exposed glycosaminoglycan-dependent adhesin J Med Microbiol 2007 56 15 22 10.1099/jmm.0.46801-0 17172511

[B43] Fadel S Eley A Differential glycosaminoglycan binding of Chlamydia trachomatis OmcB protein from serovars E and LGV J Med Microbiol 2008 57 1058 1061 10.1099/jmm.0.2008/001305-0 18719173

[B44] Fechtner T Stallmann S Moelleken K Meyer KL Hegemann JH Characterization of the interaction between the chlamydial adhesin OmcB and the human host cell J Bacteriol 2013 195 23 5323 5333 10.1128/JB.00780-13 24056107 PMC3837958

[B45] Beswick EJ Travelstead A Cooper MD Comparative studies of glycosaminoglycan involvement in Chlamydia pneumoniae and C. trachomatis invasion of host cells J Infect Dis 2003 187 1291 1300 10.1086/374056 12696009

[B46] Fudyk T Olinger L Stephens RS Selection of mutant cell lines resistant to infection by Chlamydia spp [corrected] Infect Immun 2002 70 6444 6447 10.1128/IAI.70.11.6444-6447.2002 12379725 PMC130417

[B47] Conant CG Stephens RS Chlamydia attachment to mammalian cells requires protein disulfide isomerase Cell Microbiol 2007 9 222 232 10.1111/j.1462-5822.2006.00783.x 16925789

[B48] Rosmarin DM Carette JE Olive AJ Starnbach MN Brummelkamp TR Ploegh HL Attachment of Chlamydia trachomatis L2 to host cells requires sulfation Proc Natl Acad Sci U S A 2012 109 10059 10064 10.1073/pnas.1120244109 22675117 PMC3382535

[B49] Ajonuma LC Fok KL Ho LS Chan PK Chow PH Tsang LL Wong CH Chen J Li S Rowlands DK CFTR is required for cellular entry and internalization of Chlamydia trachomatis Cell Biol Int 2010 34 593 600 10.1042/CBI20090227 20178459

[B50] Kim JH Jiang S Elwell CA Engel JN Chlamydia trachomatis co-opts the FGF2 signaling pathway to enhance infection PLoS Pathog 2011 7 e1002285 10.1371/journal.ppat.1002285 21998584 PMC3188521

[B51] Coombes BK Mahony JB Identification of MEK- and phosphoinositide 3-kinase-dependent signalling as essential events during Chlamydia pneumoniae invasion of HEp2 cells Cell Microbiol 2002 4 447 460 10.1046/j.1462-5822.2002.00203.x 12102690

[B52] Elwell CA Ceesay A Kim JH Kalman D Engel JN RNA interference screen identifies Abl kinase and PDGFR signaling in Chlamydia trachomatis entry PLoS Pathog 2008 4 e1000021 10.1371/journal.ppat.1000021 18369471 PMC2267011

[B53] Mehlitz A Banhart S Maurer AP Kaushansky A Gordus AG Zielecki J Macbeath G Meyer TF Tarp regulates early Chlamydia-induced host cell survival through interactions with the human adaptor protein SHC1 J Cell Biol 2010 190 143 157 10.1083/jcb.200909095 20624904 PMC2911661

[B54] Lane BJ Mutchler C Al Khodor S Grieshaber SS Carabeo RA Chlamydial entry involves TARP binding of guanine nucleotide exchange factors PLoS Pathog 2008 4 e1000014 10.1371/journal.ppat.1000014 18383626 PMC2279300

[B55] Jewett TJ Dooley CA Mead DJ Hackstadt T Chlamydia trachomatis tarp is phosphorylated by src family tyrosine kinases Biochem Biophys Res Commun 2008 371 339 344 10.1016/j.bbrc.2008.04.089 18442471 PMC2394672

[B56] Mehlitz A Banhart S Hess S Selbach M Meyer TF Complex kinase requirements for Chlamydia trachomatis Tarp phosphorylation FEMS Microbiol Lett 2008 289 233 240 10.1111/j.1574-6968.2008.01390.x 19016873

[B57] Reynolds DJ Pearce JH Characterization of the cytochalasin D-resistant (pinocytic) mechanisms of endocytosis utilized by chlamydiae Infect Immun 1990 58 3208 3216 2119341 10.1128/iai.58.10.3208-3216.1990 PMC313641

[B58] Schramm N Wyrick PB Cytoskeletal requirements in Chlamydia trachomatis infection of host cells Infect Immun 1995 63 324 332 7806372 10.1128/iai.63.1.324-332.1995 PMC172995

[B59] Carabeo RA Grieshaber SS Fischer E Hackstadt T Chlamydia trachomatis induces remodeling of the actin cytoskeleton during attachment and entry into HeLa cells Infect Immun 2002 70 3793 3803 10.1128/IAI.70.7.3793-3803.2002 12065523 PMC128046

[B60] Brinkworth AJ Malcolm DS Pedrosa AT Roguska K Shahbazian S Graham JE Hayward RD Carabeo RA Chlamydia trachomatis Slc1 is a type III secretion chaperone that enhances the translocation of its invasion effector substrate TARP Mol Microbiol 2011 82 131 144 10.1111/j.1365-2958.2011.07802.x 21883523 PMC3214626

[B61] Jamison WP Hackstadt T Induction of type III secretion by cell-free Chlamydia trachomatis elementary bodies Microb Pathog 2008 45 435 440 10.1016/j.micpath.2008.10.002 18984037 PMC2592499

[B62] Clifton DR Dooley CA Grieshaber SS Carabeo RA Fields KA Hackstadt T Tyrosine phosphorylation of the chlamydial effector protein Tarp is species specific and not required for recruitment of actin Infect Immun 2005 73 3860 3868 10.1128/IAI.73.7.3860-3868.2005 15972471 PMC1168552

[B63] Jewett TJ Fischer ER Mead DJ Hackstadt T Chlamydial TARP is a bacterial nucleator of actin Proc Natl Acad Sci U S A 2006 103 15599 15604 10.1073/pnas.0603044103 17028176 PMC1622868

[B64] Jewett TJ Miller NJ Dooley CA Hackstadt T The conserved Tarp actin binding domain is important for chlamydial invasion PLoS Pathog 2010 6 e1000997 10.1371/journal.ppat.1000997 20657821 PMC2904776

[B65] Jiwani S Alvarado S Ohr RJ Romero A Nguyen B Jewett TJ Chlamydia trachomatis Tarp harbors distinct G and F actin binding domains that bundle actin filaments J Bacteriol 2013 195 708 716 10.1128/JB.01768-12 23204471 PMC3562089

[B66] Jiwani S Ohr RJ Fischer ER Hackstadt T Alvarado S Romero A Jewett TJ Chlamydia trachomatis Tarp cooperates with the Arp2/3 complex to increase the rate of actin polymerization Biochem Biophys Res Commun 2012 420 816 821 10.1016/j.bbrc.2012.03.080 22465117 PMC3334425

[B67] Bullock HD Hower S Fields KA Domain analyses reveal that Chlamydia trachomatis CT694 protein belongs to the membrane-localized family of type III effector proteins J Biol Chem 2012 287 28078 28086 10.1074/jbc.M112.386904 22711538 PMC3431695

[B68] Carabeo RA Grieshaber SS Hasenkrug A Dooley C Hackstadt T Requirement for the Rac GTPase in Chlamydia trachomatis invasion of non-phagocytic cells Traffic 2004 5 418 425 10.1111/j.1398-9219.2004.00184.x 15117316

[B69] Carabeo RA Dooley CA Grieshaber SS Hackstadt T Rac interacts with Abi-1 and WAVE2 to promote an Arp2/3-dependent actin recruitment during chlamydial invasion Cell Microbiol 2007 9 2278 2288 10.1111/j.1462-5822.2007.00958.x 17501982

[B70] Subtil A Wyplosz B Balana ME Dautry-Varsat A Analysis of Chlamydia caviae entry sites and involvement of Cdc42 and Rac activity J Cell Sci 2004 117 3923 3933 10.1242/jcs.01247 15265988

[B71] Balana ME Niedergang F Subtil A Alcover A Chavrier P Dautry-Varsat A ARF6 GTPase controls bacterial invasion by actin remodelling J Cell Sci 2005 118 2201 2210 10.1242/jcs.02351 15897187

[B72] Norkin LC Wolfrom SA Stuart ES Association of caveolin with Chlamydia trachomatis inclusions at early and late stages of infection Exp Cell Res 2001 266 229 238 10.1006/excr.2001.5202 11399051

[B73] Stuart ES Webley WC Norkin LC Lipid rafts, caveolae, caveolin-1, and entry by Chlamydiae into host cells Exp Cell Res 2003 287 67 78 10.1016/S0014-4827(03)00059-4 12799183

[B74] Jutras I Abrami L Dautry-Varsat A Entry of the lymphogranuloma venereum strain of Chlamydia trachomatis into host cells involves cholesterol-rich membrane domains Infect Immun 2003 71 260 266 10.1128/IAI.71.1.260-266.2003 12496174 PMC143347

[B75] Hybiske K Stephens RS Mechanisms of Chlamydia trachomatis entry into nonphagocytic cells Infect Immun 2007 75 3925 3934 10.1128/IAI.00106-07 17502389 PMC1952008

[B76] Majeed M Kihlstrom E Mobilization of F-actin and clathrin during redistribution of Chlamydia trachomatis to an intracellular site in eucaryotic cells Infect Immun 1991 59 4465 4472 1937805 10.1128/iai.59.12.4465-4472.1991 PMC259064

[B77] Boleti H Benmerah A Ojcius DM Cerf-Bensussan N Dautry-Varsat A Chlamydia infection of epithelial cells expressing dynamin and Eps15 mutants: clathrin-independent entry into cells and dynamin-dependent productive growth J Cell Sci 1999 112 Pt 10 1487 1496 10212143 10.1242/jcs.112.10.1487

[B78] Gabel BR Elwell C van Ijzendoorn SC Engel JN Lipid raft-mediated entry is not required for Chlamydia trachomatis infection of cultured epithelial cells Infect Immun 2004 72 7367 7373 10.1128/IAI.72.12.7367-7373.2004 15557670 PMC529103

[B79] Lakadamyali M Rust MJ Zhuang X Endocytosis of influenza viruses Microbes Infect 2004 6 929 936 10.1016/j.micinf.2004.05.002 15310470 PMC2715838

[B80] Wolf K Hackstadt T Sphingomyelin trafficking in Chlamydia pneumoniae-infected cells Cell Microbiol 2001 3 145 152 10.1046/j.1462-5822.2001.00098.x 11260137

[B81] Schramm N Bagnell CR Wyrick PB Vesicles containing Chlamydia trachomatis serovar L2 remain above pH 6 within HEC-1B cells Infect Immun 1996 64 1208 1214 8606080 10.1128/iai.64.4.1208-1214.1996 PMC173905

[B82] Scidmore MA Rockey DD Fischer ER Heinzen RA Hackstadt T Vesicular interactions of the Chlamydia trachomatis inclusion are determined by chlamydial early protein synthesis rather than route of entry Infect Immun 1996 64 5366 5372 8945589 10.1128/iai.64.12.5366-5372.1996 PMC174531

[B83] Scidmore MA Fischer ER Hackstadt T Restricted fusion of Chlamydia trachomatis vesicles with endocytic compartments during the initial stages of infection Infect Immun 2003 71 973 984 10.1128/IAI.71.2.973-984.2003 12540580 PMC145390

[B84] Furtado AR Essid M Perrinet S Balana ME Yoder N Dehoux P Subtil A The chlamydial OTU domain-containing protein ChlaOTU is an early type III secretion effector targeting ubiquitin and NDP52 Cell Microbiol 2013 15 12 2064 2079 10.1111/cmi.12171 23869922

[B85] Clausen JD Christiansen G Holst HU Birkelund S Chlamydia trachomatis utilizes the host cell microtubule network during early events of infection Mol Microbiol 1997 25 441 449 10.1046/j.1365-2958.1997.4591832.x 9302007

[B86] Grieshaber SS Grieshaber NA Hackstadt T Chlamydia trachomatis uses host cell dynein to traffic to the microtubule-organizing center in a p50 dynamitin-independent process J Cell Sci 2003 116 3793 3802 10.1242/jcs.00695 12902405

[B87] Mital J Hackstadt T Diverse requirements for SRC-family tyrosine kinases distinguish chlamydial species MBio 2011 doi: 10.1128/mBio.00031-11 10.1128/mBio.00031-11 PMC3063380 21427287

[B88] Heinzen RA Scidmore MA Rockey DD Hackstadt T Differential interaction with endocytic and exocytic pathways distinguish parasitophorous vacuoles of Coxiella burnetii and Chlamydia trachomatis Infect Immun 1996 64 796 809 8641784 10.1128/iai.64.3.796-809.1996 PMC173840

[B89] Al Younes HM Rudel T Meyer TF Characterization and intracellular trafficking pattern of vacuoles containing Chlamydia pneumoniae in human epithelial cells Cell Microbiol 1999 1 237 247 10.1046/j.1462-5822.1999.00024.x 11207556

[B90] Capmany A Damiani MT Chlamydia trachomatis intercepts Golgi-derived sphingolipids through a Rab14-mediated transport required for bacterial development and replication PloS one 2010 5 e14084 10.1371/journal.pone.0014084 21124879 PMC2989924

[B91] Rzomp KA Scholtes LD Briggs BJ Whittaker GR Scidmore MA Rab GTPases are recruited to chlamydial inclusions in both a species-dependent and species-independent manner Infect Immun 2003 71 5855 5870 10.1128/IAI.71.10.5855-5870.2003 14500507 PMC201052

[B92] Moorhead AM Jung JY Smirnov A Kaufer S Scidmore MA Multiple host proteins that function in phosphatidylinositol-4-phosphate metabolism are recruited to the chlamydial inclusion Infect Immun 2010 78 1990 2007 10.1128/IAI.01340-09 20231409 PMC2863499

[B93] Moorhead AR Rzomp KA Scidmore MA The Rab6 effector Bicaudal D1 associates with Chlamydia trachomatis inclusions in a biovar-specific manner Infect Immun 2007 75 781 791 10.1128/IAI.01447-06 17101644 PMC1828475

[B94] Leiva N Capmany A Damiani MT Rab11-family of interacting protein 2 associates with chlamydial inclusions through its Rab-binding domain and promotes bacterial multiplication Cell Microbiol 2013 15 114 129 10.1111/cmi.12035 23006599

[B95] Carabeo RA Mead DJ Hackstadt T Golgi-dependent transport of cholesterol to the Chlamydia trachomatis inclusion Proc Natl Acad Sci U S A 2003 100 6771 6776 10.1073/pnas.1131289100 12743366 PMC164522

[B96] Hackstadt T Scidmore MA Rockey DD Lipid metabolism in Chlamydia trachomatis-infected cells: directed trafficking of Golgi-derived sphingolipids to the chlamydial inclusion Proc Natl Acad Sci U S A 1995 92 4877 4881 10.1073/pnas.92.11.4877 7761416 PMC41810

[B97] Heuer D Lipinski AR Machuy N Karlas A Wehrens A Siedler F Brinkmann V Meyer TF Chlamydia causes fragmentation of the Golgi compartment to ensure reproduction Nature 2009 457 731 735 10.1038/nature07578 19060882

[B98] Rejman Lipinski A Heymann J Meissner C Karlas A Brinkmann V Meyer TF Heuer D Rab6 and Rab11 regulate Chlamydia trachomatis development and golgin-84-dependent Golgi fragmentation PLoS Pathog 2009 5 e1000615 10.1371/journal.ppat.1000615 19816566 PMC2752117

[B99] Moore ER Fischer ER Mead DJ Hackstadt T The chlamydial inclusion preferentially intercepts basolaterally directed sphingomyelin-containing exocytic vacuoles Traffic 2008 9 2130 2140 10.1111/j.1600-0854.2008.00828.x 18778406 PMC2951019

[B100] Moore ER Mead DJ Dooley CA Sager J Hackstadt T The trans-Golgi SNARE syntaxin 6 is recruited to the chlamydial inclusion membrane Microbiology 2011 157 830 838 10.1099/mic.0.045856-0 21109560 PMC3081085

[B101] Pokrovskaya ID Szwedo JW Goodwin A Lupashina TV Nagarajan UM Lupashin VV Chlamydia trachomatis hijacks intra-Golgi COG complex-dependent vesicle trafficking pathway Cell Microbiol 2012 14 656 668 10.1111/j.1462-5822.2012.01747.x 22233276 PMC3330190

[B102] Beatty WL Trafficking from CD63-positive late endocytic multivesicular bodies is essential for intracellular development of Chlamydia trachomatis J Cell Sci 2006 119 350 359 10.1242/jcs.02733 16410552

[B103] Cocchiaro JL Kumar Y Fischer ER Hackstadt T Valdivia RH Cytoplasmic lipid droplets are translocated into the lumen of the Chlamydia trachomatis parasitophorous vacuole Proc Natl Acad Sci U S A 2008 105 9379 9384 10.1073/pnas.0712241105 18591669 PMC2453745

[B104] Cox JV Naher N Abdelrahman YM Belland RJ Host HDL biogenesis machinery is recruited to the inclusion of Chlamydia trachomatis-infected cells and regulates chlamydial growth Cell Microbiol 2012 14 1497 1512 10.1111/j.1462-5822.2012.01823.x 22672264 PMC3443303

[B105] Elwell CA Jiang S Kim JH Lee A Wittmann T Hanada K Melancon P Engel JN Chlamydia trachomatis co-opts GBF1 and CERT to acquire host sphingomyelin for distinct roles during intracellular development PLoS Pathog 2011 7 e1002198 10.1371/journal.ppat.1002198 21909260 PMC3164637

[B106] Mital J Hackstadt T Role for the SRC family kinase Fyn in sphingolipid acquisition by chlamydiae Infect Immun 2011 79 4559 4568 10.1128/IAI.05692-11 21896774 PMC3257913

[B107] Li Z Chen C Chen D Wu Y Zhong Y Zhong G Characterization of fifty putative inclusion membrane proteins encoded in the Chlamydia trachomatis genome Infect Immun 2008 76 2746 2757 10.1128/IAI.00010-08 18391011 PMC2423075

[B108] Bannantine JP Stamm WE Suchland RJ Rockey DD Chlamydia trachomatis IncA is localized to the inclusion membrane and is recognized by antisera from infected humans and primates Infect Immun 1998 66 6017 6021 9826388 10.1128/iai.66.12.6017-6021.1998 PMC108764

[B109] Bannantine JP Griffiths RS Viratyosin W Brown WJ Rockey DD A secondary structure motif predictive of protein localization to the chlamydial inclusion membrane Cell Microbiol 2000 2 35 47 10.1046/j.1462-5822.2000.00029.x 11207561

[B110] Toh H Miura K Shirai M Hattori M In silico inference of inclusion membrane protein family in obligate intracellular parasites chlamydiae DNA Res 2003 10 9 17 10.1093/dnares/10.1.9 12693550

[B111] Heinz E Rockey DD Montanaro J Aistleitner K Wagner M Horn M Inclusion membrane proteins of Protochlamydia amoebophila UWE25 reveal a conserved mechanism for host cell interaction among the Chlamydiae J Bacteriol 2010 192 5093 5102 10.1128/JB.00605-10 20675479 PMC2944539

[B112] Lutter EI Martens C Hackstadt T Evolution and conservation of predicted inclusion membrane proteins in chlamydiae Comp Funct Genomics 2012 2012 362104 22454599 10.1155/2012/362104 PMC3290821

[B113] Dehoux P Flores R Dauga C Zhong G Subtil A Multi-genome identification and characterization of chlamydiae-specific type III secretion substrates: the Inc proteins BMC Genomics 2011 12 109 10.1186/1471-2164-12-109 21324157 PMC3048545

[B114] Subtil A Parsot C Dautry-Varsat A Secretion of predicted Inc proteins of Chlamydia pneumoniae by a heterologous type III machinery Mol Microbiol 2001 39 792 800 10.1046/j.1365-2958.2001.02272.x 11169118

[B115] Fields KA Mead DJ Dooley CA Hackstadt T Chlamydia trachomatis type III secretion: evidence for a functional apparatus during early-cycle development Mol Microbiol 2003 48 671 683 10.1046/j.1365-2958.2003.03462.x 12694613

[B116] Suchland RJ Rockey DD Bannantine JP Stamm WE Isolates of Chlamydia trachomatis that occupy nonfusogenic inclusions lack IncA, a protein localized to the inclusion membrane Infect Immun 2000 68 360 367 10.1128/IAI.68.1.360-367.2000 10603409 PMC97142

[B117] Alzhanov D Barnes J Hruby DE Rockey DD Chlamydial development is blocked in host cells transfected with Chlamydophila caviae incA BMC Microbiol 2004 4 24 10.1186/1471-2180-4-24 15230981 PMC459217

[B118] Delevoye C Nilges M Dautry-Varsat A Subtil A Conservation of the biochemical properties of IncA from Chlamydia trachomatis and Chlamydia caviae: oligomerization of IncA mediates interaction between facing membranes J Biol Chem 2004 279 46896 46906 10.1074/jbc.M407227200 15316015

[B119] Delevoye C Nilges M Dehoux P Paumet F Perrinet S Dautry-Varsat A Subtil A SNARE protein mimicry by an intracellular bacterium PLoS Pathog 2008 4 e1000022 10.1371/journal.ppat.1000022 18369472 PMC2265411

[B120] Muschiol S Bailey L Gylfe A Sundin C Hultenby K Bergstrom S Elofsson M Wolf-Watz H Normark S Henriques-Normark B A small-molecule inhibitor of type III secretion inhibits different stages of the infectious cycle of Chlamydia trachomatis Proc Natl Acad Sci U S A 2006 103 14566 14571 10.1073/pnas.0606412103 16973741 PMC1566191

[B121] Verbeke P Welter-Stahl L Ying S Hansen J Hacker G Darville T Ojcius DM Recruitment of BAD by the Chlamydia trachomatis vacuole correlates with host-cell survival PLoS Pathog 2006 2 e45 10.1371/journal.ppat.0020045 16710454 PMC1463014

[B122] Cortes C Rzomp KA Tvinnereim A Scidmore MA Wizel B Chlamydia pneumoniae inclusion membrane protein Cpn0585 interacts with multiple Rab GTPases Infect Immun 2007 75 5586 5596 10.1128/IAI.01020-07 17908815 PMC2168330

[B123] Rzomp KA Moorhead AR Scidmore MA The GTPase Rab4 interacts with Chlamydia trachomatis inclusion membrane protein CT229 Infect Immun 2006 74 5362 5373 10.1128/IAI.00539-06 16926431 PMC1594829

[B124] Mital J Miller NJ Fischer ER Hackstadt T Specific chlamydial inclusion membrane proteins associate with active Src family kinases in microdomains that interact with the host microtubule network Cell Microbiol 2010 12 1235 1249 10.1111/j.1462-5822.2010.01465.x 20331642 PMC2923664

[B125] Derre I Swiss R Agaisse H The lipid transfer protein CERT interacts with the Chlamydia inclusion protein IncD and participates to ER-Chlamydia inclusion membrane contact sites PLoS Pathog 2011 7 e1002092 10.1371/journal.ppat.1002092 21731489 PMC3121800

[B126] Dumoux M Clare DK Saibil HR Hayward RD Chlamydiae assemble a pathogen synapse to hijack the host endoplasmic reticulum Traffic 2012 13 1612 1627 10.1111/tra.12002 22901061 PMC3533787

[B127] Lutter EI Barger AC Nair V Hackstadt T Chlamydia trachomatis Inclusion Membrane Protein CT228 Recruits Elements of the Myosin Phosphatase Pathway to Regulate Release Mechanisms Cell Rep 2013 3 1921 1931 10.1016/j.celrep.2013.04.027 23727243 PMC3700685

